# Case Report: Analysis of antimicrobial therapy and pharmaceutical care of mixed *Aspergillus* and *Mycobacterium tuberculosis* infection following renal transplantation: a case study

**DOI:** 10.3389/fmed.2026.1968738

**Published:** 2026-09-30

**Authors:** Jiao Zhang, Yue Zhao, Yang Peng, Xin Zheng

**Affiliations:** 1 Department of Pharmacy, Dianjiang People’s Hospital of Chongqing, Chongqing, China; 2 Department of Pharmacy, the First Affiliated Hospital of Army Medical University, Chongqing, China

**Keywords:** aspergillus, immunosuppressants, mycobacterium tuberculosis, pharmaceutical care, renal transplantation

## Abstract

Invasive infection is a significant complication following renal transplantation. Due to long-term immunosuppressive therapy, renal transplant recipients are at high risk for fungal infections and tuberculosis, which are also major causes of mortality in this population. Triazole drugs, as first-line agents for invasive fungal infections, can cause fluctuations in the blood levels of immunosuppressants due to drug-drug interactions. Furthermore, the concomitant use of antituberculosis drugs further complicates the balance between maintaining immunosuppression and anti-infective therapy, often presenting a challenge to clinical decision-making. This article discusses the role of the clinical pharmacist in the treatment of a renal transplant recipient who developed concurrent pulmonary tuberculosis and *aspergillus* infection. By recommending initial regimens, adjusting doses during outpatient follow-ups, and assessing adverse drug reactions, the clinical pharmacist provided systematic pharmaceutical services. This collaboration assisted physicians in developing a personalized treatment plan, ultimately achieving favorable therapeutic outcomes for the patient.

## 1 Introduction

Renal transplantation remains the most ideal renal replacement therapy for patients with end-stage chronic renal failure (1, 2). The use of immunosuppressive agents' post-transplantation is a crucial therapeutic strategy for improving long-term graft survival. Nevertheless, balancing immunosuppression and anti-infective efficacy to enhance both recipient and long-term graft survival remains a pivotal challenge for clinicians (3, 4). This case reported a renal transplant recipient who exhibited a severe infection with *Mycobacterium tuberculosis* (MTB) and *Aspergillus*. The clinical pharmacist helped develop a balanced strategy for anti-infective and immunosuppressive therapy. This strategy was informed by therapeutic drug monitoring (TDM) and a comprehensive assessment of multiple drug interactions. The objective of this case study was to provide a reference strategy for the rational use of medications in the management of analogous complex infection scenarios.

## 2 Case presentation

A 21-year-old woman was admitted to the department of nephrology on July 23, 2025, complaining of cough and back pain for 4 days following renal transplantation 6 years prior. The patient underwent right heterologous renal transplantation in 2019. She has been regularly taking immunosuppressive medications postoperatively, with serum creatinine levels fluctuating between 90 and 100 μmol/L. Current regimen: prednisone 5 mg qd, mycophenolate mofetil (MMF) 375 mg bid, cyclosporine A (CsA) 75 mg AM and 50 mg PM. The patient did not have diabetes, coronary heart disease, or other infectious diseases. On admission physical examination, the transplanted renal area in the right lower abdomen was non-tender. Pulmonary infection was suspected.

On the first day of admission, laboratory tests, inflammatory markers, and pulmonary imaging were completed: white-cell count (WBC) was 9.9 × 10^9^/L, procalcitonin (PCT) was 0.06 ng/mL. Blood CsA concentration was 112.2 ng/mL, creatinine (CR) was 137.8 μmol/L, and uric acid (UA) was 338 μmol/L. Chest computed tomography (CT) revealed left pleural effusion, partial atelectasis of the left lower lobe with inflammatory changes, and segmental bronchiectasis with infection in the left upper lobe. Given the patient's current cough symptoms, empirical antimicrobial therapy with ceftriaxone was initiated.

On day 6, the patient developed a fever of 38.5 °C. Additionally, the results of interferon-gamma release assays were positive. A repeat chest CT scan showed a slight increase in left pleural effusion, as well as new multifocal infections in the right lung. Due to worsening pulmonary infection symptoms, the antimicrobial regimen was adjusted to meropenem, and a bronchoscopy with lung lavage was performed.

On day 7, 16S rRNA sequencing of the bronchoalveolar lavage fluid revealed *Aspergillus* (sequence 2) and MTB complex (sequence 281). The clinical pharmacist recommended isavuconazole as the antifungal agent and a rifampin-free anti-tuberculosis (anti-TB) regimen. The anti-TB regimen was initiated with levofloxacin, isoniazid (INH), pyrazinamide (PZA), and ethambutol (EMB).

On day 10, the CR level rose to 155.2 μmol/L and the CsA concentration increased to 259.8 ng/mL, prompting a dosage adjustment of CsA to 25 mg every 12 h.

On day 12, the patient's cough had significantly improved without fever or chest pain. The CT scans showed reduced left pleural effusion, lessened consolidation and atelectasis in the left lung, and diminished multiple infections in the right lung. The patient was discharged today with instructions for outpatient follow-up. Discharge medication regimen was prednisolone 5 mg once daily, CsA 25 mg every 12 h, MMF 375 mg every 12 h.

One week later (August 13), outpatient follow-up revealed persistently elevated CsA and creatinine levels, reaching 184.5 ng/mL and 120.5 μmol/L, respectively. The pharmacist recommended reducing CsA to 25 mg once daily and adding vitamin B6 to prevent optic neuritis. The patient returned on September 17, with CsA concentration at 54.8 ng/mL and creatinine at 115 μmol/L. Due to the significant reduction in the dosage of CsA, a detailed medication history was immediately recorded. It was retroactively discovered that another hospital had initiated rifapentine treatment without adjusting the dosage of CsA. Due to persistently low CsA levels, the dosage was increased to 25 mg every 12 h. At the October 16 follow-up, CsA concentration rebounded to 159.5 ng/mL, while creatinine increased to 140.4 μmol/L. Changes in renal function and CsA concentrations during this process are shown in Table 1. To maintain overall immunosuppressive balance, the pharmacist advised adjusting the MMF dosage. Additionally, the patient provided a CT report from another hospital indicating that the pulmonary inflammation had largely resolved. Therefore, it was recommended to adjust the anti-TB regimen to levofloxacin combined with INH. The patient discontinued isavuconazole in November, and the cumulative duration of antifungal treatment reached 10 weeks. Regimen adjustments and CT images are detailed in Figures 1, 2.

**Table 1:** Serial changes in serum creatinine, uric acid, and CsA concentrations.

Time	CR (umol/L)	UA (umol/L)	Concentration of CsA (ng/mL)
2025-5-19	96.5	285	139.4
2025-7-23	137.8	338	112.2
2025-7-29	102.3	229	142.1
2025-8-4	155.2	288	259.8
2025-8-13	120.5	591	184.5
2025-9-2	85.3	546	62.2
2025-9-17	115	667	54.8
2025-10-16	140.40	606	159.5

**Figure 1:**
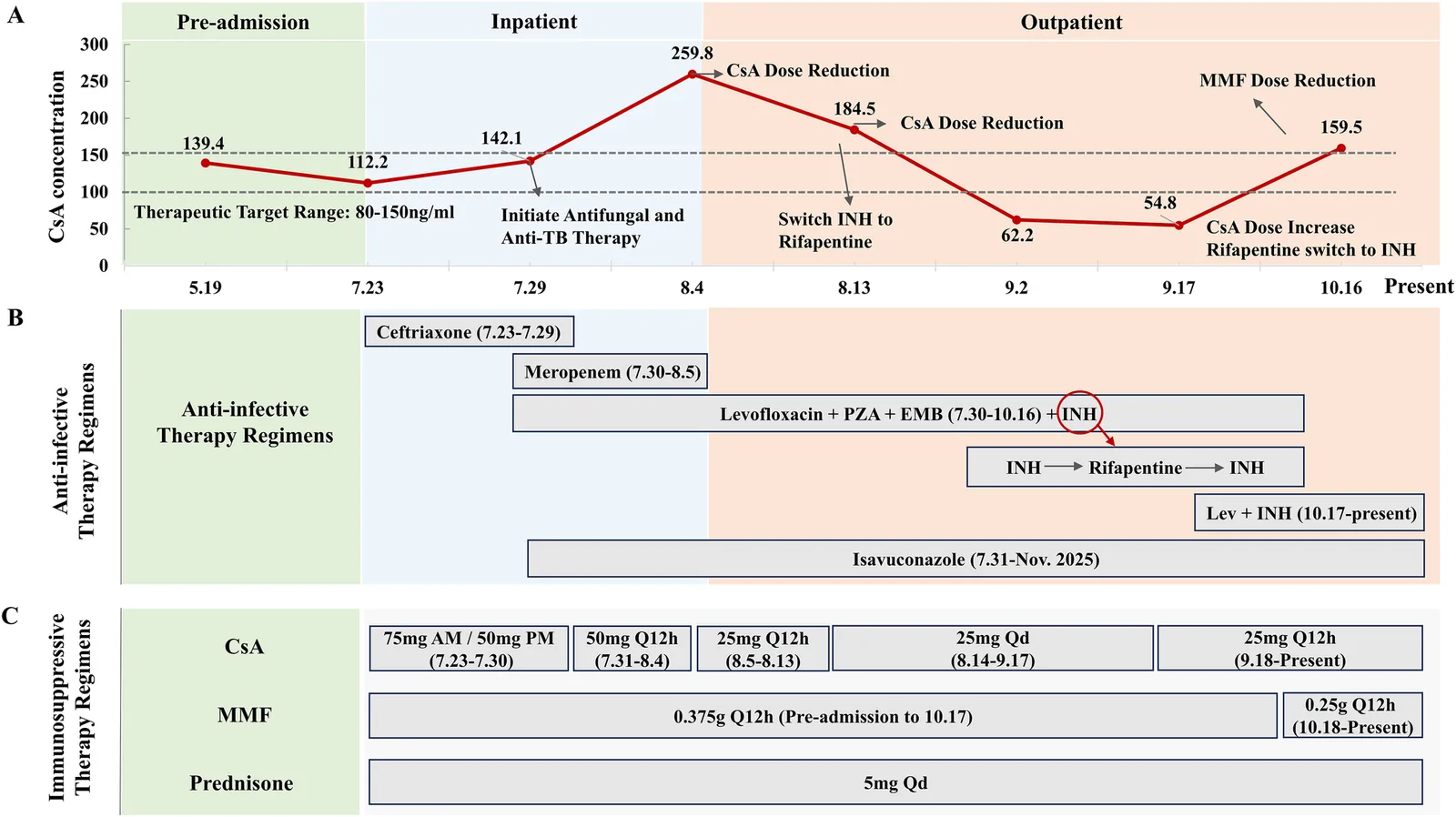
Changes in CsA plasma concentrations and treatment regimens during the treatment course. **(A)** Serial CsA plasma concentrations and major therapeutic adjustments. **(B)** Anti-infective therapy regimens. **(C)** Immunosuppressive therapy regimens.

**Figure 2:**
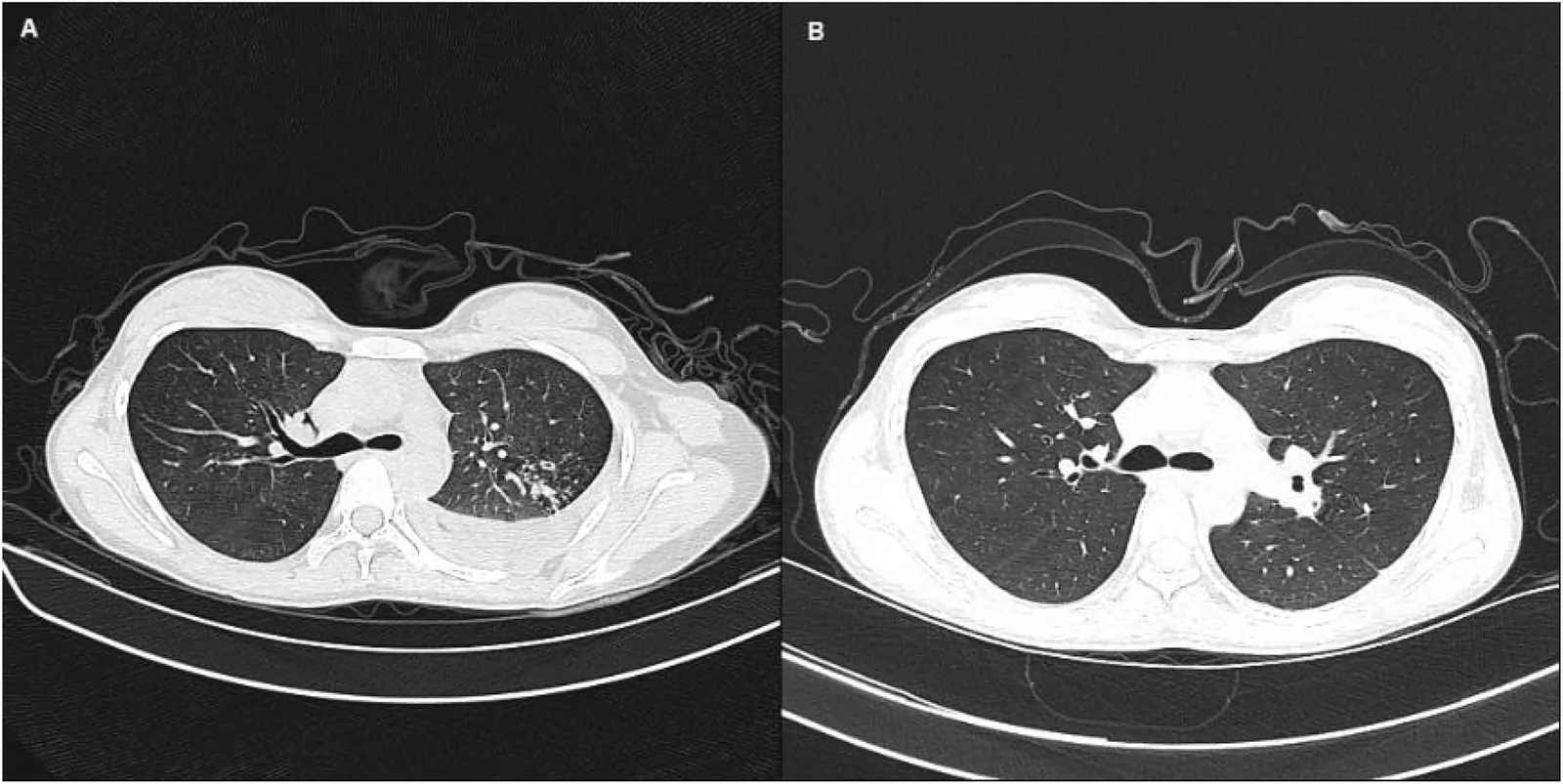
Chest CT images before and after antimicrobial therapy. **(A)** Representative CT slice showing active mixed pulmonary infection at initial presentation on July 31, 2025. **(B)** Follow-up CT scan on February 27, 2026, demonstrating marked radiological improvement and lesion resolution after 10 weeks of treatment.

## 3 Discussion

Organ transplant recipients constitute the highest-risk group for fungal infections, which are characterized by insidious onset, diagnostic challenges, and high mortality rates (5, 6). Their clinical presentation differs from that of the general population, the onset is often subtle with a minimal inflammatory response, and typical symptoms such as high fever and chills are uncommon. Imaging findings are diverse and non-specific, frequently presenting multiple nodules, consolidation, or bronchopneumonia-like changes that are difficult to distinguish from bacterial or tuberculous infections (7). In this case, the patient initially presented with only a cough and shoulder pain, a normal temperature, and imaging that revealed pleural effusion and atelectasis. The subsequent confirmation of a mixed *Aspergillus* and MTB illustrates the non-specific nature of infections in transplant recipients.

For renal transplant recipients, the development of infection can be considered a manifestation of immune imbalance, resulting from a multitude of factors, including transplant-related, medication-related, and host-related factors. The one-year cumulative incidence of invasive fungal infection in renal transplant recipients is 1.3%, with a median onset time of 6 months post-surgery and 43% of cases diagnosed within the first 6 months (8, 9). Sustained intensity immunosuppression is the core risk factor. The triple regimen not only suppresses T-cell function but also weakens antifungal immunity. The patient is a young woman with no other comorbidities, who presented with mildly elevated creatinine levels prior to admission. However, a review of medical history revealed that the CsA concentrations over the preceding year ranged from 115 to 166.8 ng/mL. According to the Chinese Guidelines, when renal transplant recipients have been on triple therapy for >12 months, the target trough concentration for CsA is recommended to be in the range of 80–150 ng/mL (10). This elevated level of immunosuppression significantly increased susceptibility to infection.

The core pharmaceutical challenge in this case was balancing anti-infective efficacy with the intensity of immunosuppression. For pulmonary *Aspergillus* infection, the pharmacist recommended isavuconazole as the first-line agent based on its comparable efficacy to voriconazole but with superior safety (11). In terms of drug interactions, based on the pharmacokinetic studies of midazolam (a CYP3A4 probe substrate), voriconazole, posaconazole, and itraconazole all increased the AUC of midazolam by ≥5 times, while isavuconazole increased the AUC of midazolam by ≥2 times but <5 times belonging to a moderate inhibitor. Moreover, its inhibitory effect on CYP3A4 is significantly weaker, and the risk of interaction with immunosuppressants is reduced (12). Given that this patient requires long-term CsA therapy, and to reduce the risk of nephrotoxicity or rejection reactions caused by significant fluctuations in immunosuppressant plasma concentrations, the pharmacist preferred to recommend isavuconazole as the antifungal treatment drug. However, isavuconazole still caused a significant increase in the CsA blood concentration.

During anti-TB therapy, to avoid significant reduction in CsA levels by rifampin, the clinical pharmacist initially selected a rifampin-free regimen (13). However, during a follow-up visit at another hospital, the regimen was adjusted to replace isoniazid with rifapentine, leading to persistently low CsA levels. Therefore, we recommended reverting to the original medication regimen.

Through repeated TDM and multiple dose adjustments, CsA levels were ultimately restored to the target range. Furthermore, the patient no longer reported discomfort related to tuberculosis or fungal infections. In solid organ transplantation, when a severe infection occurs, clinicians must rapidly reduce the dosage of immunosuppressants to facilitate the clearance of pathogen. However, this reduction in immunosuppressant dosage, combined with the activation of the innate immune system and systemic inflammatory cascades triggered by the severe infection, leaves the transplanted organ in a highly vulnerable state. Consequently, these patients face a significantly increased risk of acute graft rejection. The need to simultaneously manage life-threatening infections and maintain graft survival underscores the critical importance of TDM and multidisciplinary collaboration in optimizing outcomes.

## 4 Conclusion

Complex drug interactions in post-renal transplant mixed infections cause fluctuations in immunosuppressant concentrations, making TDM-guided individualized dosing essential. Clinical pharmacists ensure the balance between anti-infective and immunosuppressive therapies through continuous monitoring and optimized regimens, providing pharmaceutical care for complex combination therapy settings.

## Data Availability

The clinical data supporting the conclusions of this article will be made available by the authors upon reasonable request. The 16S rRNA sequencing was performed by a third-party clinical laboratory as part of routine clinical care, and the raw sequencing data are not held by the authors.
